# Fractionating Boost Dose in Postoperative Breast Radiotherapy

**DOI:** 10.7759/cureus.84561

**Published:** 2025-05-21

**Authors:** Georgios Moschos, Georgios Plataniotis

**Affiliations:** 1 Department of Radiation Oncology, Aristotle University of Thessaloniki, Thessaloniki, GRC

**Keywords:** clinical radiobiology, hypofractionated boost, hypofractionated breast radiotherapy, tumor bed boost, whole breast irradiation

## Abstract

Mild hypofractionation in postoperative breast radiotherapy (RT) is now widely accepted as the preferred treatment, especially after the publication of the START trials in the United Kingdom. A boost to postoperative whole-breast irradiation (WBI) is indicated in selected patients to further reduce the risk of local recurrence. However, the exact dose and fractionation of radiation boost are subject to clinical debate. In the present work, we calculated the biologically effective doses (BEDs, linear-quadratic model) of WBI and boost RT prescribed in the most cited clinical trials to suggest an acceptable trial-based dose and fractionation for boost RT. WBI of a BED value of 65-75 Gy_3.5 _is required, and a boost-BED of 15-19 Gy_3.5 _is considered adequate. Longer boost RT schedules with a BED value of higher than 20 Gy_3.5 _do not offer additional benefits and might result in a higher risk for long-term post-radiation effects and waste of resources.

## Introduction and background

Whole-breast irradiation (WBI) is indicated for the management of early breast cancer in women who have undergone breast-conserving surgery (BCS) [1]. Traditionally, breast cancer radiotherapy (RT) has been delivered in conventionally fractionated schedules of 1.8-2.0 Gy per fraction (fx), up to a total dose of 45-50.4 Gy over five to seven weeks.

In selected women, an extra radiation dose ("boost") to the tumor bed - the area most likely to contain residual microscopic disease - is delivered to further reduce the risk of local recurrence. The boost typically refers to an additional 10-16 Gy, targeting a reduced volume and administered after or integrated within WBI [2]. The well-known trial by the European Organization for Research and Treatment of Cancer (EORTC) [3] demonstrated the benefit of a boost using 16 Gy in eight fractions, although alternative fractionation schedules have also been used [4-8].

In recent years, hypofractionated radiotherapy (HRT) for WBI has become the standard of care. However, insufficient data are available to evaluate a "hypofractionated boost," and clinical oncologists have a rather intuitive clinical approach to prescribing dose and fractionation for boost RT. Overall, the timing, dose, and fractionation vary, and a standardized approach has yet to be established. Moreover, several guidelines (e.g., ASTRO, ESTRO) do not provide harmonized recommendations regarding boost use or technique [2,9].

The biologically effective dose (BED), derived from the isoeffective linear-quadratic (LQ) model, is a key radiobiological concept used to compare different fractionation schemes by incorporating total dose, fraction size, and tissue sensitivity, expressed as the α/β ratio, which reflects a tissue’s fractionation sensitivity, i.e. its response to dose per fraction [10-12]. It is particularly useful in the hypofractionation era, where clinical schedules vary significantly in terms of time, dose, and fractionation and must be biologically normalized to inform local control and toxicity outcomes.

The purpose of this narrative review and basic (elementary) clinical radiobiology analysis was to explore the trends in clinical practice regarding boost fractionation, given that the issue of WBI fractionation has been largely resolved by the START and FAST-FORWARD trials (see below). We also intended to show that boost doses in the range of 16 Gy in 8 fractions are an overtreatment, and there is no need to consider it any longer, given the outcomes of other reputable published clinical trials and prospective studies. See also our analysis in reference [12], published in the Red Journal. In this review, we further highlight which patient subgroups (e.g., younger age, high-risk histopathologic features) are most likely to benefit from the addition of a tumor bed boost to WBI.

## Review

Methods

Method of Literature Search

This narrative review summarizes current evidence on tumor bed boost in conventional and hypofractionated WBI for early-stage breast cancer. A non-systematic search of PubMed/MEDLINE and ClinicalTrials.gov (2000-March 2025) used terms including “breast cancer”, “radiotherapy”, “boost”, and “hypofractionation”. Priority was given to English-language phase II-III RCTs, meta-analyses, guidelines (ASTRO, ESTRO, NCCN), and large observational studies reporting on local control or toxicity, provided BED could be calculated using the LQ model. Studies focusing on postoperative boost were prioritized, while DCIS, younger patients, and nodal irradiation were included when relevant. Case reports, non-English articles, and studies lacking extractable clinical endpoints were excluded.

Studies were grouped into six themes: (1) conventional boost trials, (2) international guidelines, (3) hypofractionated WBI trials, (4) hypofractionated boost trials, (5) boost in hypofractionated WBI, and (6) BED modeling for WBI and boost.

Data were extracted independently by two reviewers using a standardized template, including study design, patient characteristics, radiation details, boost parameters, BED, and outcomes. Discrepancies were resolved by consensus. No formal risk of bias tool or meta-analysis was applied; results are presented descriptively.

Radiobiological Modeling Approach

To facilitate comparison across different fractionation schemes and boost strategies, the BED was calculated using the LQ model, with an α/β ratio of 3.5 Gy for breast cancer, as determined in the START trials for breast cancer cells [10-12]. The BED formula is:

BED = D(1+d/(α⁄β)),

where D is the total dose and d is the dose per fraction. Repopulation of tumor cells was not considered, given the uncertainty of when exactly it starts in the case of postoperative breast RT and how fast it is given following operation and adjuvant systemic treatments (chemotherapy, hormones) preceding RT. However, we must mention that Haviland et al. [13], after a post-hoc analysis of START trials, calculated the repopulation rate at 0.6 Gy/day for breast cancer postoperative RT. However, they did not define its "kick-off" time. Adding the two weeks in a five-week RT schedule implies a wasted RT dose of 0.6 Gy/day x 14 days = 8 Gy. That lowers the 50 Gy dose in the standard fractionation RT schedule to 42 Gy, which approaches the equivalent of 40 Gy/15 fractions. It could be suggested that exponential cancer cell growth may be uniformly distributed throughout RT time for the postoperative cases. If that happens, repopulation is expected to affect all the above-mentioned RT schedules by the same proportion.

Repopulation may impact tumor control, particularly in longer RT schedules; however, this does not appear to be the case in breast cancer, as it is in squamous cell carcinomas of the head and neck - especially now, with the clinical implementation of one- to three-week RT schedules. We have to note that this is not a clinical radiobiology analysis; therefore, sensitivity analysis was not performed. BED values should be interpreted with caution and considered only as indicators of the relative biological effectiveness of the various RT schedules used in clinical practice.

Results

Conventional Boost Trials

Multiple randomized trials have evaluated the benefit of a tumor bed boost following conventional WBI (50 Gy in 25 fractions) after BCS for early-stage breast cancer, focusing on local control, toxicity, and cosmetic outcomes.

The EORTC trial [3], with a median follow-up of 17.2 years, demonstrated that administration of a 16 Gy boost in eight fractions (16 Gy/8 fxs) significantly reduced the 20-year cumulative incidence of ipsilateral breast tumor recurrence compared to no boost (12.0% vs. 16.4%; HR 0.65, 95% CI: 0.53-0.80; p < 0.0001), with the largest absolute benefit observed in women <50 years or with high-grade tumors. However, no statistically significant difference in 20-year overall survival (OS) was observed (59.7% with boost vs. 61.1% without; HR 1.05, 95% CI: 0.93-1.18; p = 0.39). The rate of severe fibrosis was significantly higher in the boost group (5.2% vs. 1.8%; p < 0.0001), especially among patients aged ≤40 and 41-50 years.

Building on this evidence, a related EORTC trial [4], with 10.4 years’ median follow-up, compared a 26 Gy versus 10 Gy boost in patients with close or positive margins. Although the higher dose was associated with numerically improved 10-year local control (10.8% vs. 17.5%), the difference was not statistically significant (HR = 0.83, 95% CI: 0.43-1.57; p > 0.1). Importantly, the 26 Gy arm experienced a markedly higher rate of severe fibrosis (14.4% vs. 3.3%), illustrating the dose-toxicity trade-off without a clear oncologic benefit.

Supporting this pattern, the Budapest boost trial [5], with a median follow-up of 5.3 years, randomized patients to WBI with or without boost and showed improved five-year local control in the boost group (92.7% vs. 84.9%; HR = 0.42, 95% CI: 0.17-1.02; p = 0.049). Boost was delivered via either 16 Gy electron therapy or 12-14.25 Gy HDR brachytherapy. Local control was comparable between techniques (94.2% vs. 91.4%; p = 0.74), but brachytherapy was associated with significantly more fibrosis (17.3% vs. 1.9%; p = 0.008). Overall, grade 2-3 late toxicity was more frequent in the boost arm (17.3% vs. 7.8%; p = 0.03), while cosmetic outcomes were similar. On multivariate analysis, age <40, positive margins, and high mitotic index were independent predictors of local recurrence.

Similarly, the Lyon trial [6], with a median follow-up of 5.2 years, evaluated a 10 Gy/5 fxs electron boost versus no boost, demonstrating significantly reduced recurrence (HR = 0.46, 95% CI: 0.22-0.95) but again higher late toxicity (12.4% vs. 5.9%) and no difference in overall survival (p = 0.24). This reinforces the consistent finding across trials that boost improves local control at the cost of increased late effects.

Shifting focus toward cosmetic outcomes, the St. George and Wollongong trial [7], with a five-year median follow-up, evaluated whether adding a 16 Gy/8 fxs boost to a reduced WBI dose of 45 Gy/25 fxs (boost group), compared to standard WBI alone of 50 Gy/25 fxs (control group), could preserve cosmetic outcomes without compromising tumour control. The boost group showed superior excellent/good cosmesis (79% vs. 68%; OR = 1.77, 95% CI: 1.1-2.8; p = 0.016), while maintaining equivalent local control.

The Young-Boost trial [8], a more recent study, with a median follow-up of 11.7 years, investigated dose escalation (26 Gy vs. 16 Gy boost) in women ≤50 years, all of whom received conventional WBI (50 Gy/25 fxs). The higher boost dose significantly reduced 10-year local recurrence (2.8% vs. 4.4%; HR = 0.61, 95% CI: 0.39-0.96; p = 0.032), but increased rates of fibrosis (48% vs. 27%; p < 0.001), with no difference in overall survival, reaffirming the benefit-toxicity balance in younger patients.

International Boost Guidelines

International consensus guidelines support the use of a tumor bed boost in selected patients with early-stage breast cancer, although recommendations between ASTRO and ESTRO are not fully aligned. The 2018 ASTRO guideline [2], which remains the most recent ASTRO recommendation specifically focused on boost in breast RT, strongly recommends a boost for women with invasive breast cancer who are aged ≤50 years regardless of tumour grade, as well as for those aged 51-70 years with high-grade tumours or positive surgical margins. For patients with ductal carcinoma in situ (DCIS), ASTRO also advises a boost in cases of age ≤50 years, high-grade disease, or close (<2 mm) or positive margins.

Alternatively, the 2022 ESTRO guideline [9] recommends a more individualized approach, stratifying patients into low-, intermediate-, and high-risk categories. A boost is advised only in intermediate- or high-risk cases, based on factors such as age <45 years, high-grade histology, lymphovascular invasion, positive or close margins, extensive intraductal component, and unfavorable molecular subtype (e.g., triple-negative or HER2-positive). In low-risk patients, a boost is discouraged due to limited benefit and potential for increased toxicity.

These discrepancies between ASTRO and ESTRO recommendations highlight the need for further international harmonization of criteria regarding boost indication, to ensure consistency in clinical practice and optimize patient outcomes.

Hypofractionated WBI Trials

Over the past two decades, hypofractionated radiotherapy (HRT) has become a standard for whole breast and chest wall irradiation in early-stage breast cancer, based on strong clinical evidence. Breast cancer clonogens exhibit similar fraction sensitivity to late-responding normal tissues, as reflected by low α/β values in the LQ model [12]. Among the landmark randomized trials, the UK START and Ontario studies [14-17] played a central role in establishing the efficacy and safety of HRT after breast-conserving surgery. Table 1 summarizes the pivotal trials and their RT regimens [14-25]. The TROG 07.01 trial [21] further demonstrated that hypofractionation is feasible and well tolerated in patients with ductal carcinoma in situ (DCIS).

**Table 1:** Clinical trials testing hypofractionation in postoperative breast radiotherapy. The most important clinical trials have evaluated hypofractionated schedules in postoperative breast radiotherapy, either with or without a tumor bed boost [14–25]. Biologically effective dose (BED) values were calculated for each radiotherapy (RT) schedule using the linear-quadratic (LQ) formula, assuming an α/β of 3.5 Gy and excluding the time (repopulation) correction factor [10,11]. † In the FAST-Forward trial [23], a boost was administered to approximately 25% of patients across all treatment arms. Of these, only 5% received 16 Gy in eight fractions, while the remaining 20% received 10 Gy in five fractions. ‡ In the IMPORT trials [24,25], RT was delivered either to the whole breast (WBI), the quadrant containing the resected tumor (PBI-1), or exclusively to the tumor bed (PBI-2). A simultaneous integrated boost (SIB) approach was employed in most arms, maintaining a consistent total treatment duration. In the IMPORT HIGH trial [25]; however, the boost of 16 Gy in eight fractions was given sequentially, thereby extending the overall treatment time from 18 to 30 days. Since recurrence sites were not specified within the high- or low-dose regions, only the highest BED value was considered in dose–response calculations. Abbreviations: WBI: whole-breast irradiation, PBI: partial-breast irradiation, BED: biologically effective dose, LRR: local recurrence rate. Note: Fractionation is represented as total dose/fractions/duration in weeks (e.g., 50/25/5 = 50 Gy in 25 fractions over five weeks).

Name of the trial	Dose per fraction (Gy)	% of patients who had a boost	BED (Gy3.5)	Boost BED (Gy3.5)	Total BED (Gy3.5)	Number of patients	Five-year LRR (%)	10-year LRR (%)
Start P		75%				1410		
50/25/5	2.0	14 Gy/7	78.6	22	100.6	470	7.9	12.1
42.9/13/5	3.3	14 Gy/7	83.3	22	105.3	466	7.1	9.6
39/13/5	3.0	14 Gy/7	72.4	22	94.4	474	9.1	14.8
Start A		60%				2236		
50/25/5	2.0	10 Gy/5	78.6	15.7	94.3	749	3.4	6.7
41.6/13/5	3.2	10 Gy/5	79.6	15.7	95.3	750	3.1	5.6
39/13/5	3.0	10 Gy/5	72.4	15.7	94.4	737	4.4	8.1
Start B		42-43%				2215		
50/25/5	2.0	10 Gy/5	78.6	15.7	94.3	1105	3.3	5.2
40/15/3	2.67	10 Gy/5	70.6	15.7	86.3	1110	1.9	3.8
Ontario		No boost				1234		
50/25/5	2.0	N/A	78.6	N/A	78.6	612	3.2	6.7
42.5/16/3.2	2.66	N/A	75	N/A	75	622	2.8	6.2
Fast		No boost				915		
50/25/5	2.0	N/A	78.6	N/A	78.6	302	0.7	0.7
30/5/5	6.0	N/A	81.4	N/A	81.4	308	1.0	1.4
28.5/5/5	5.7	N/A	75	N/A	75	305	0.4	1.7
Fast forward^†^		25%				4096		
40/15/3	2.67	10 Gy/5 (20%), 16 Gy/8 (5%)	70.6	15.7 (20%), 25.1 (5%)	86.3 (20%), 95.7 (5%)	1361	2.1	N/A
27/5/1	5.4	10 Gy/5 (20%), 16 Gy/8 (5%)	68.7	15.7 (20%), 25.1 (5%)	84.4 (20%), 93.8 (5%)	1367	1.7	N/A
26/5/1	5.2	10 Gy/5 (20%), 16 Gy/8 (5%)	64.6	15.7 (20%), 25.1 (5%)	80.3 (20%), 89.7 (5%)	1368	1.4	N/A
Import low^‡^		N/A				2016		
40/15/3 WBI	2.67	N/A	70.6	N/A	70.6	674	1.1	N/A
36/15/3 WBI, 40/15/3 PBI	2.4, 2.67	N/A	60.7, 70.6	N/A	60.7, 70.6	673	0.2	N/A
40/15/3 PBI	2.67	N/A	70.6	N/A	70.6	669	0.5	N/A
Import high^‡^		N/A				2617		
40/15/3 WBI	2.67	16 Gy/8	70.6	25	95.6	871	1.9	N/A
36/15/3 WBI,40/15/3 PBI-1, 48/15/3 PBI-2	2.4, 2.67, 3.2	N/A	60.7, 70.6, 91.9	N/A	60.7, 70.6, 91.9	874	2	N/A
36/15/3 WBI, 40/15/3 PBI-1, 53/15/3 PBI-2	2.4, 2.67, 3.53	N/A	60.7, 70.6, 105	N/A	60.7, 70.6, 105	872	3.2	N/A
Chinese		100%				734		
50/25/5	2.0	10 Gy/5	78.6	15.7	94.3	366	2.0	N/A
43.5/15	2.9	8.7 Gy/3	79.5	15.9	95.4	368	1.2	N/A
MDACC, 22% DCIS		100%				287		
50/25/5	2.0	10 Gy/5	78.6	15.7	94.3	149	2	N/A
42.5/16	2.66	10 Gy/4	74.9	17	92	138	1	N/A
DBCG-HYPO, 13% DCIS		23%				1854		
50/25/5	2.0	10 Gy/5	78.6	15.7	94.3	937	N/A	3.3
40/15/3	2.67	10 Gy/5	70.6	15.7	86.3	917	N/A	3.0
TROG(07.01), 100% DCIS		50%				1608		
50/25/5	2.0	16 Gy/8	78.6	25	103.6	803	5	N/A
42.5/16	2.66	16 Gy/8	74.9	25	100	831	5	N/A

Moving beyond standard hypofractionated regimens, the FAST and FAST-FORWARD trials [22,23] assessed ultra-hypofractionated WBI, delivering 28.5-30 Gy in five once-weekly fractions and 26 Gy in five daily fractions over one week, respectively. Both trials demonstrated comparable tumor control with favorable toxicity profiles. In parallel efforts to optimize RT delivery, the IMPORT trials [24,25] investigated partial-breast irradiation (PBI) and the simultaneous integrated boost (SIB) technique, confirming the effectiveness of these approaches in maintaining local control while reducing toxicity. Hypofractionated RT to the nodal areas has also shown non-inferior safety outcomes compared to conventional fractionation, supporting broader application beyond the breast alone [26].

These findings are further supported by a 2021 meta-analysis by Gu et al. [27], which demonstrated equivalent tumor control and treatment-related toxicity between hypofractionated and conventional RT. Based on this cumulative evidence, HRT has become the standard approach for whole breast, chest wall, and regional nodal irradiation. This shift is reflected in current guidelines from the American Society for Radiation Oncology (ASTRO) and the European Society for Radiotherapy and Oncology (ESTRO), which recommend hypofractionation regardless of patient age, tumor stage, or use of chemotherapy [2, 9]. Similarly, the 2025 edition of the National Comprehensive Cancer Network (NCCN) guidelines also endorses HRT for WBI.

Hypofractionated Boost Trials

With hypofractionated WBI increasingly established as standard care, randomized evidence has begun to explore whether hypofractionated boost regimens can achieve similar oncologic efficacy with lower toxicity.

A Chinese trial [19] compared conventional WBI + boost (50 + 10 Gy) with a hypofractionated protocol (43.5 Gy/15 fxs WBI + 8.7 Gy/3 fxs boost). With a median follow-up of 6.1 years, five-year local relapse rates were comparable (1.2% vs. 2.0%; HR = 0.62, 95% CI: 0.20-1.88), while acute toxicity was significantly lower in the hypofractionated group (3% vs. 8%; p = 0.019), suggesting that shorter regimens may achieve equivalent tumor control with better tolerability.

Similarly, a recent MD Anderson trial [20] randomized patients to conventional (50 + 10 Gy) or hypofractionated (42.5 Gy/16 fxs + 10 Gy/4 fxs) WBI plus boost. At three years, local recurrence-free survival was 99% in both arms. Hypofractionation was associated with a lower rate of patient-reported adverse cosmesis (8.2% vs. 13.6%; p = 0.002) and significantly fewer acute toxicities (47% vs. 78%; p < 0.01), without differences in late toxicity or survival. These findings support the incorporation of boost into contemporary hypofractionated regimens, offering an optimal balance between efficacy and patient-reported outcomes.

Boost in Hypofractionated WBI

The most up-to-date guideline by ASTRO specifies that the choice to include a boost should not be contingent upon selecting the whole breast fractionation scheme [2]. However, insufficient data are available to evaluate the addition of a boost to hypofractionated WBI.

To this end, a post-hoc analysis of the UK START trials found no statistically significant difference in local-regional relapse between patients who received a boost and those who did not, regardless of whether they underwent standard or hypofractionated WBI (HR: 0.99, 95% CI: 0.76-1.29) [13]. This suggests that the positive effect of a boost on local control may be independent of the fractionation schedule. Despite these findings, real-world practice patterns tell a different story. Several national and institutional studies have reported reduced boost usage in the hypofractionated setting compared to conventional fractionation [28-31]. These observations raise concerns about potential undertreatment and deviation from guideline-based care.

BED Modeling for WBI and Boost

BEDs were calculated for the principal clinical trials discussed in this review, focusing on testing hypofractionation in postoperative breast RT (with or without boost), as presented in Table 1. Analysis of the trials suggests, for the WBI, a BED value of 65-75 Gy_3.5_ is required to reduce local recurrence rates to acceptably low levels. Under the above-mentioned indications, adding a boost with an equivalent BED of 15-18 Gy_3.5_ is required. Boost schedules resulting in a BED above 20 Gy_3.5_ should generally be avoided, as they may constitute overtreatment, increasing the likelihood of long-term complications such as fibrosis, telangiectasia, and adverse cosmetic outcomes.

Interestingly, there is a trend of higher prescribed BEDs in older trials and paradoxically higher recurrence rates. The local recurrence rates show a trend for reduction in newer trials, despite the lower prescribed BEDs, probably because of the improvements in both surgical techniques and systemic treatments. Our previous report calculated a total BED of 90 Gy_4_ to maximize tumor control probability (TCP) [12]. That corresponds to a BED of about 92-94 Gy_3.5_ (depending on the size of the dose per fraction) if a lower α/β ratio of 3.5 Gy was used for the radiobiological calculations.

Discussion

Hypofractionation in postoperative breast RT has become a highly acceptable schedule for most patients. The LQ-based clinical radiobiology of the START trials has calculated an α/β ratio of 3.5 Gy for breast cancer cells [11]. Therefore, three-week RT schedules yield BED values between 65 and 75 Gy_3.5_ and have become the standard for postoperative WBI. Shorter and more hypofractionated RT schedules (such as 26 Gy in 5 fractions over one week) are also gaining popularity, although a longer time might be needed until clinical data mature.

In the present work, we reviewed the recurrence rates reported in the most cited clinical trials addressing WBI, hypofractionation, fast hypofractionation, partial breast RT, and breast RT boost. We calculated the Biologically Effective Doses (BEDs) corresponding to each fractionation schedule, for both WBI and boost RT, in order to roughly define a clinically safe BED range for the breast RT boost. This analysis is aligned with previous work we have published [12], which aimed to determine a WBI dose-response relationship.

We acknowledge the limitations inherent in this non-systematic review, including potential publication bias, heterogeneity in outcome definitions (e.g., cosmetic grading), and variability across study designs, patient populations, and RT techniques. No formal risk of bias assessment (e.g., Cochrane RoB2) or statistical modeling was performed. Consequently, findings are presented descriptively, which limits their statistical generalizability but remains consistent with the narrative and biologically oriented focus of this review. Furthermore, the present review did not include studies from low- and middle-income countries (LMICs), limiting the global generalizability of the findings. This underscores the need for region-specific, prospective research.

Late toxicities like fibrosis are dose-dependent and influenced by BED, particularly when a boost is used. BED estimation aids in selecting regimens that balance tumor control with normal tissue safety. Based on this, different fractionation schemes may be selected depending on individual risk. For example, when there is a high risk of excessive fibrosis, a standard regimen such as 45 Gy/25 fxs (1.8 Gy/fx) over five weeks may be considered, yielding a WBI BED of 72 Gy_3.5_ [32]. If a boost is indicated in such cases, 10 Gy in 5 fractions can be added, resulting in a total BED of approximately 87.7 Gy_3.5_. In cases where a simultaneous integrated boost is preferred, one commonly tested regimen involves 40 Gy to the whole breast (2.67 Gy/fx) and 48 Gy to the tumor bed (3.2 Gy/fx), which corresponds to a BED of around 92 Gy_3.5_ to the tumor bed. These examples illustrate how treatment selection can be guided by BED thresholds to optimize both efficacy and long-term safety

Our review and analysis support the establishment of BED-based reference ranges for breast RT boost, particularly within the context of hypofractionated WBI. A total boost BED of 15-18 Gy_3.5_ appears both effective and safe, as evidenced by commonly used regimens such as 10 Gy/5 fxs or 10.67 Gy/4 fxs. On the contrary, boost regimens resulting in a BED above 20 Gy_3.5_ should generally be avoided, as they may increase the risk of late toxicity without providing significant additional benefit.

## Conclusions

In patients at low risk for local recurrence, for whom a boost is not indicated, WBI alone may be sufficient. In such cases, HRT schedules such as 40 Gy/15 fxs over three weeks or 26 Gy/5 fxs delivered in one week, in appropriately selected patients, are recommended, producing BEDs of 70.6 and 64.6 Gy_3.5_, respectively, according to the LQ model.

For patients at higher risk of local recurrence, for whom a boost is indicated, options such as 10 Gy/4 fxs, 10 Gy/5 fxs, or 10.67 Gy/4 fxs may be employed, corresponding to BEDs of 15.7, 17, and 19 Gy_3.5_, respectively. The resulting total BED for WBI combined with a boost ranges between 80.3 and 87.6 Gy_3.5_, offering effective tumor control while maintaining acceptable toxicity profiles.

For patients considered at high risk of developing late radiation effects, standard fractionation schedules are preferred. A regimen of 45 Gy/25 fxs over five weeks provides a total BED of 72 Gy_3.5_. In such cases, if a boost is indicated, a dose of 10 Gy/5 fxs may be appropriate to minimize the risk of late toxicity.

Boost regimens with a BED exceeding 20 Gy_3.5_ do not appear to confer additional clinical benefit and may increase the likelihood of long-term adverse effects. Therefore, such regimens should be avoided to reduce unnecessary toxicity and optimize treatment efficiency.

## References

[1] Darby S, McGale P, Correa C (2011). Effect of radiotherapy after breast-conserving surgery on 10-year recurrence and 15-year breast cancer death: meta-analysis of individual patient data for 10,801 women in 17 randomised trials. Lancet.

[2] Smith BD, Bellon JR, Blitzblau R (2018). Radiation therapy for the whole breast: executive summary of an American Society for Radiation Oncology (ASTRO) evidence-based guideline. Pract Radiat Oncol.

[3] Bartelink H, Maingon P, Poortmans P (2015). European Organisation for Research and Treatment of Cancer Radiation Oncology and Breast Cancer Groups. Whole-breast irradiation with or without a boost for patients treated with breast-conserving surgery for early breast cancer: 20-year follow-up of a randomised phase 3 trial. Lancet Oncol.

[4] Poortmans PM, Collette L, Bartelink H (2008). The addition of a boost dose on the primary tumour bed after lumpectomy in breast conserving treatment for breast cancer. A summary of the results of EORTC 22881-10882 "boost versus no boost" trial. Cancer Radiother.

[5] Polgár C, Fodor J, Orosz Z (2002). Electron and high-dose-rate brachytherapy boost in the conservative treatment of stage I-II breast cancer first results of the randomized Budapest boost trial. Strahlenther Onkol.

[6] Romestaing P, Lehingue Y, Carrie C (1997). Role of a 10-Gy boost in the conservative treatment of early breast cancer: results of a randomized clinical trial in Lyon, France. J Clin Oncol.

[7] Hau E, Browne LH, Khanna S (2012). Radiotherapy breast boost with reduced whole-breast dose is associated with improved cosmesis: the results of a comprehensive assessment from the St. George and Wollongong randomized breast boost trial. Int J Radiat Oncol Biol Phys.

[8] Bosma S, van Werkhoven E, Bartelink H (20241). Young boost randomized phase III trial of high vs low boost radiation in young breast cancer patients: 10-year results. Eur J Cancer.

[9] Meattini I, Becherini C, Boersma L (2022). European Society for Radiotherapy and Oncology Advisory Committee in Radiation Oncology Practice consensus recommendations on patient selection and dose and fractionation for external beam radiotherapy in early breast cancer. Lancet Oncol.

[10] Fowler JF (1989). The linear-quadratic formula and progress in fractionated radiotherapy. Br J Radiol.

[11] Yarnold J (2019). Changes in radiotherapy fractionation-breast cancer. Br J Radiol.

[12] Plataniotis GA, Dale RG (2009). Biologically effective dose-response relationship for breast cancer treated by conservative surgery and postoperative radiotherapy. Int J Radiat Oncol Biol Phys.

[13] Haviland J, Owen J, Dewar J (2013). The UK Standardisation of Breast Radiotherapy (START) trials of radiotherapy HRT for treatment of early breast cancer: 10-year follow-up results of two randomised controlled trials. Lancet Oncol.

[14] Yarnold J, Ashton A, Bliss J (2005). Fractionation sensitivity and dose response of late adverse effects in the breast after radiotherapy for early breast cancer: long-term results of a randomised trial. Radiother Oncol.

[15] Bentzen SM, Agrawal RK, Aird EG (2008). The UK Standardisation of Breast Radiotherapy (START) Trial A of radiotherapy hypofractionation for treatment of early breast cancer: a randomised trial. Lancet Oncol.

[16] Bentzen SM, Agrawal RK, Aird EG (2008). The UK Standardisation of Breast Radiotherapy (START) Trial B of radiotherapy hypofractionation for treatment of early breast cancer: a randomised trial. Lancet.

[17] Whelan TJ, Pignol JP, Levine MN (2010). Long-term results of hypofractionated radiation therapy for breast cancer. N Engl J Med.

[18] Offersen BV, Alsner J, Nielsen HM (2020). Hypofractionated versus standard fractionated radiotherapy in patients with early breast cancer or ductal carcinoma in situ in a randomized phase III trial: the DBCG HYPO trial. J Clin Oncol.

[19] Wang SL, Fang H, Hu C (2020). Hypofractionated versus conventional fractionated radiotherapy after breast-conserving surgery in the modern treatment era: a multicenter, randomized controlled trial from China. J Clin Oncol.

[20] Shaitelman SF, Lei X, Thompson A (2018). Three-year outcomes with hypofractionated versus conventionally fractionated whole breast irradiation: results of a randomized, non-inferiority clinical trial. J Clin Oncol.

[21] Chua B, Link E, Kunkler I (2022). Radiation doses and fractionation schedules in non-low-risk ductal carcinoma in situ in the breast (BIG 3-07/TROG 07.01): a randomised, factorial, multicentre, open-label, phase 3 study. Lancet.

[22] Brunt AM, Haviland JS, Sydenham M (2020). Ten-year results of FAST: a randomized controlled trial of 5-fraction whole-breast radiotherapy for early breast cancer. J Clin Oncol.

[23] Murray Brunt A, Haviland JS, Wheatley DA (2020). Hypofractionated breast radiotherapy for 1 week versus 3 weeks (FAST-Forward): 5-year efficacy and late normal tissue effects results from a multicentre, non-inferiority, randomised, phase 3 trial. Lancet.

[24] Coles CE, Griffin CL, Kirby AM (2017). Partial-breast radiotherapy after breast conservation surgery for patients with early breast cancer (UK IMPORT LOW trial): 5-year results from a multicentre, randomised, controlled, phase 3, non-inferiority trial. Lancet.

[25] Coles C, Haviland J, Kirby A (2023). Dose-escalated simultaneous integrated boost radiotherapy in early breast cancer (IMPORT HIGH): a multicentre, phase 3, non-inferiority, open-label, randomised controlled trial. Lancet.

[26] Owen JR, Ashton A, Bliss JM (2006). Effect of radiotherapy fraction size on tumour control in patients with early-stage breast cancer after local tumour excision: long-term results of a randomised trial. Lancet Oncol.

[27] Gu L, Dai W, Fu R (2021). Comparing hypofractionated with conventional fractionated radiotherapy after breast-conserving surgery for early breast cancer: a meta-analysis of randomized controlled trials. Front Oncol.

[28] Stokes WA, Jackson MW, Kounalakis N (2017). Disparities and patterns of care in boost utilization for early-stage breast cancer. Int J Radiat Oncol.

[29] Zhong J, Kandula S, Liu X (2016). Patterns of radiation therapy boost administration in 356,160 breast cancer patients treated with hypofractionated versus conventional whole-breast irradiation. Int J Radiat Oncol.

[30] Hasan Y, Waller J, Yao K, Chmura SJ, Huo D (2017). Utilization trend and regimens of hypofractionated whole breast radiation therapy in the United States. Breast Cancer Res Treat.

[31] Hoopes DJ, Kaziska D, Chapin P, Weed D, Smith BD, Hale ER, Johnstone PA (2012). Patient preferences and physician practice patterns regarding breast radiotherapy. Int J Radiat Oncol Biol Phys.

[32] Koulis TA, Phan T, Olivotto IA (2015). Hypofractionated whole breast radiotherapy: current perspectives. Breast Cancer (Dove Med Press).

